# Conformal Prediction in Clinical Medical Sciences

**DOI:** 10.1007/s41666-021-00113-8

**Published:** 2022-01-28

**Authors:** Janette Vazquez, Julio C. Facelli

**Affiliations:** Department of Biomedical Informatics and Clinical and Translational Science Institute, The University of Utah, Salt Lake City, UT 84108 USA

**Keywords:** Artificial intelligence in medicine, Conformal Prediction, Predictive analytics, Uncertainty quantification

## Abstract

The use of machine learning (ML) and artificial intelligence (AI) applications in medicine has attracted a great deal of attention in the medical literature, but little is known about how to use Conformal Predictions (CP) to assess the accuracy of individual predictions in clinical applications. We performed a comprehensive search in SCOPUS® to find papers reporting the use of CP in clinical applications. We identified 14 papers reporting the use of CP for clinical applications, and we briefly describe the methods and results reported in these papers. The literature reviewed shows that CP methods can be used in clinical applications to provide important insight into the accuracy of individual predictions. Unfortunately, the review also shows that most of the studies have been performed in isolation, without input from practicing clinicians, not providing comparisons among different approaches and not considering important socio-technical considerations leading to clinical adoption.

## Introduction

The use of machine learning (ML) and artificial intelligence (AI) applications in medicine has attracted a great deal of attention in the medical literature. While we cannot provide a comprehensive list of commentaries and viewpoints published in the most influential medical journals, the following references provide a general overview of the field [1–7]. Several issues arise from this body of literature. The most pertinent to this review is the lack of methods enabling uncertainty quantification (UQ), generalizability, and reproducibility of clinical machine learning. The current state of the art for evaluating the performance of clinical predictive models is to provide values that measure overall global performance, like predictive value and the area under the curve [5, 8, 9]. However, while these global properties are fundamental in assessing the potential clinical impact that such a model may have when applied to a large patient population, they do not provide any information about the confidence in individual predictions. It is noteworthy that the model prediction for different individuals will have very different intervals of confidence because the distribution of the predictors does not follow normal distributions. Therefore, the quality of prediction will depend on the topology of the feature space in the proximity of the next prediction. Likely, the predictions for individuals in regions of smooth variation and well represented in the training feature space will have much larger confidence intervals than those for individuals from regions less represented or from more roughed landscapes in the training feature space. This is particularly concerning when using predictive analytics for individuals of underserved populations that systematically are excluded from the training sets used in parametrizing predictive models [10, 11]. Because medical decisions based on ML predictive clinical models should be made for each individual patient and not for a population, determining confidence intervals for individual predictions of these models is critical if these models will be adopted in clinical settings. A promising approach to provide uncertainty for each individual prediction is Conformal Prediction (CP) [12, 13]. In this paper, we provide a succinct discussion of CP methods followed by a discussion of published CP applications to clinical medical sciences.

## Conformal Prediction

Conformal Prediction (CP) [12, 13] has been proposed as one avenue to address the issue of providing levels of reliability for individual predictions. As argued in ref. [14], CP is also appealing because it can be explained in an intuitive manner. The reasoning is that for a given new test instance (*x*_n_), the predicted class label (*y*_n_) will be a reliable prediction when (*x*_n_) is similar to the training instances, while it will be less reliable when the reverse is true. This is a concept that both non-computer scientists and statistic experts can grasp.

To apply Conformal Prediction to a predictive or machine learning model, a calibration or training set and a non-conformity measure to quantify how “strange” a label *y* is for a given instance *x* are necessary [21]. In this section, we give a brief description of CP (with more details available in refs. [19, 20]).

Given a training set (*x*_1_, *y*_1_), …, (*x*_*n* − 1_, *y*_*n* − 1_), where each *x*_*i*_ ∈ *X* is a vector of attributes for example *i* and *y*_*i*_ ∈ *Y* is the classification or label of that example, and a new unclassified example *x*_*n*_, the task of CP is to state something about the confidence in each possible classification. CP assigns each one of the possible labels to the new example *x*_*n*_ one by one and measures how likely it is for the set of examples (*x*_*n*_, *y*) to have been generated independently from the same probability distribution. The ideal case occurs when the predicted label conforms with the rest of the levels in the sequence, indicating that we can be confident in the prediction [20].

In classification algorithms, standard non-conformity measures are often like uncertainty measures such as the least confidence score (1—the predicted probability). For example, a non-conformity measure for a classification task could be the ratio of distance to the nearest neighbors with the same label by the distance to the nearest neighbor with different labels. Reporting prediction sets at a certain significance level (or several) is one way of presenting the prediction produced by the conformal predictor. Another way is to report the point prediction, the credibility, and the confidence. A high confidence (close to 1) means that there is no likely alternative to the point prediction and a low (close to 0) credibility means that the point prediction is unlikely [20]. In regression, the efficiency of a conformal predictor is determined by the size of the predicted confidence regions. The prediction set is often an interval of values, and a natural measure of efficiency of such prediction is simply the length of the interval, with the smaller the length of the interval is the better it is for performance [20].

Conformal Prediction can be used in combination with any machine learning algorithm, and no additional parameterization is required except for the selection of the non-conformity measure. Another main advantage of conformal predictors is their validity. CPs are valid if the assumption of exchangeability is fulfilled and if the randomness assumption is fulfilled [26].

**Fig. 1.**
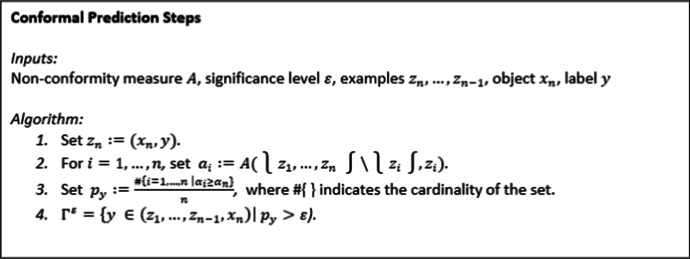
Archetypical pseudocode for CP implementation

Conformal Prediction (CP) in computer science literature contains many articles where CP has been applied to various fields such as forensics, biometrics, and facial recognition, or where approaches towards CP aim to reduce computational complexity or improve confidence values [15]. Variations of CP are described in multiple papers [16, 17]. However, the overall implementation of CP tends to be relatively similar. First, a non-conformity measure is chosen, the machine learning model is then trained, the trained model is applied to the test set or sequence, and the non-conformity is evaluated. Reliable predictions can then be identified to give the significance and confidence levels and evaluate the validity and efficiency of the generated conformal predictor. In Fig. 1 we provide a pseudocode of the typical manner that CP methods are implemented.

First, the non-conformity measure, *A*, is defined and calculated. It is usually based on a traditional machine learning algorithm, which can be referred to as the underlying algorithm of the CP, to measure how strange or “non-conforming” each example is for the rest of the examples in the same set [22]. This measure assigns a numerical score *α*_*i*_ to each example (*x*_*i*_, *y*_*i*_) indicating how different it is from all other examples, as shown in step 2 of the CP algorithm in Fig. 1. Training the underlying algorithm as the training set generates a prediction rule

$${\alpha}_i:= A\left\{\left({x}_1,{y}_1\right),\dots, \left({x}_{i-1,},{y}_{i-1}\right),\left({x}_{i+1},{y}_{i+1}\right),\dots, \left({x}_n,{y}_n\right),\left({x}_i,{Y}_i\right)\right\}.$$

After we consider a hypothesis *y*_*n*_ = *y* and calculate the corresponding non-conformity scores *α*_1_, …, *α*_*n*_, we can compare *α*_*n*_with the other *α*_*i*_s by calculating:

$${P}_y=\frac{\#\left\{i=1,\dots, n\ \right|{\alpha}_i\ge {\alpha}_n\Big\}}{n}.$$

This ratio is called the *p* value associated with *Y*, which lies between *l/n* and 1, where #{ } indicates the cardinality of the set. Here, we look at the fraction of the examples least different from others and form a prediction region consisting of *y* not among the most out of place when added to the bag of old examples [22]. For this calculation, if the *p* value of a given label is under a low threshold (0.10), this indicates the label is highly unlikely as the sets will only be generated at the most 10% of the time. Labels with a *p* value under a very low significance level can be excluded [22].

CP methods that were found in the literature are reviewed here (see the “Literature Search” section and Table 1) and reported as used in medical clinical applications included Inductive Conformal Predictors (ICP), Mondrian Conformal Predictors (MCP), Label-Conditional Mondrian Conformal Predictors (LCMCP), Dynamic Conformal Predictors (DCP), Inductive Confidence Machine (ICM), and Generalized Learning Vector Quantizer (GLVQ). A brief description of these methods is given in the following subsections. For further reading and details on these individual methods, the reader can refer to the work of Vovk et al. [19] or the individual papers where these methods were mentioned (Table 1).

**Table 1.** Methods used in Conformal Prediction Studies for medical applications

First author	Classification method(s) ^a^	Conformal Prediction method(s)
Pereira [14, 20]	KNN, Naïve Bayes, and ensemble classifiers	Mondrian predictors and CP with scaling
Papadopoulos [21–23]	ANN	Mondrian predictors, LCMCP
Alnemer [24]	SVM, DT, KNN, ANN	Non-conformity score^b^
Devetyarov [25]	Linear rules	Mondrian predictors
Lambrou [26–28]	Rule-based, GA, SVM	Based on the evolved decision rule after prediction
Luo [29]	SVM	Dynamic Conformal Prediction
Schleif [30]	SNG	GLVQ ^c^
Balasubramanian [31]	SVM	Computed with respect to both class levels
Bellotti [32]	SVM	Inductive Confidence Machine

^a^*KNN* K-nearest neighbor, *SVM* support vector machine, *DT* decision tree, *ANN* artificial neural networks, *GA* genetic algorithms, *SNG* supervised neural gas

^b^NC score calculated by comparing the distance of the new prediction point to all records in the training set that have the same label to its distance to the rest of the training set

^c^*GLVQ* Generalized Learning Vector Quantizer

### Inductive Conformal Prediction (ICP)

The original CP technique requires training the underlying algorithm for each possible classification of every new test example, often making it computationally complex and inefficient. Inductive Conformal Predictors (ICPs) was created to address this issue by training the underlying algorithm only once, making it more computationally efficient than previous CP methods for algorithms with long training times. ICPs split the training set into two smaller sets, often referred to as the proper training set and a calibration set. The proper training set is used to train the underlying algorithm to generate a prediction rule, and the calibration set is used to calculate the *p* value of each possible classification [22].

### Mondrian Conformal Prediction (MCP)

In Mondrian Conformal Prediction (MCP), each label or class is treated separately and the confidence in the assignment of a given instance to the classes considered is evaluated independently. Using the predictions for the calibration set, each class generates a list of non-conformity scores. For example, in medical diagnosis, certain patients may be easier to correctly classify than others which would result in an overall error rate higher in certain groups of patients that may be harder to classify or an error rate lower in patients easier to classify [26]. MCP guarantees the error rate within these groups by splitting training sets into categories and setting a significance for each category, with the categories either based on features or a combination of features. MCP also compares the non-conformity score only among those within the same category and not across all training sets, making it a good choice for imbalanced data sets [26]. Label-Conditional Mondrian Conformal Prediction (LCMCP) is a special case of MCP in which the category of each example is determined by its label or classification [24].

### Inductive Confidence Machine (ICM)

The Confidence Machine is a relatively new classification and prediction framework that originates from work on the algorithmic randomness theory and is based on a given underlying induction rule [22]. Computational efficiency is almost as good as the underlying algorithm for ICMs, and although there is some loss in the quality of confidence, the loss is often not too serious. The outputs of the ICM also have a clearer probabilistic interpretation [18].

### Generalized Learning Vector Quantizer (GLVQ)

Generalized Learning Vector Quantizer (GLVQ) and variants are successful prototype-based learning algorithms [31]. A common property among these variants is the existence of distances used in the cost function to optimize the prototype positions. To transform GLVQ into a conformal predictor, a non-conformity measure is determined. For prototype-based networks, a measure of non-conformity for a given sample is the sample margin as the distance of the data point to the closest prototype with the same class normalized by the distance of this item to the closest prototype with an alternative class [31].

### Dynamic Conformal Prediction (DCP)

Dynamic Conformal Prediction (DCP) was designed in ref. [30]. CP’s time complexity and lack of adaptation make it unsuitable for many real applications. To overcome these shortcomings, DCP was proposed. It provides multiple advantages over CP, such as dealing with multi-testing samples and a new form of confidence based on the idea of conformity score. It was designed to provide higher accuracy and a lower computational complexity. In DCP, the set of training samples is iteratively updated after a pre-specified time. The system continuously brings in new training samples and deserts older training samples, essential for time-varying systems where the system or data may change over time. After processing using the base classifier, the prediction gives the label and the confidences in prediction of the testing samples. However, DCP and CP differ in terms of confidence prediction. DCP only utilizes credibility, not confidence, and instead a new confidence measure is designed and used in DCP. The new form of confidence proposed for DCP is not influenced by the distribution of data points, making it useful for imbalanced data sets [30].

Variations of Conformal Prediction are described in multiple papers [16, 17]. These papers show the confidence values obtained by CPs, their usefulness in practice for various applications, and how their algorithm can often perform better than standard CP algorithms. Two books also show milestones in the related CP literature. One is *Algorithmic Learning in a Random World*, written by Vovk et al. [18], which explains the theoretical fundamentals of CP. A more recent book, *Conformal Prediction for reliable machine learning* by Balasubramanian et al. [19], shows the practical applications and adaptations of CP to real-world problems.

## Literature Search

**Fig. 2.**
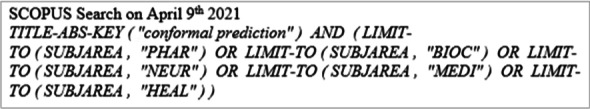
SCOPUS Query used in this review

We have searched for Conformal Prediction articles with medical science applications on April 2021 using the below query in SCOPUS (see Fig. 2).

The SCOPUS search was followed by manual selection by the authors on the basis of the titles, abstracts, and full text. The authors rejected papers using CP in medical applications such as toxicology and drug discovery, animal models, image analysis, and “neuro computing.” These were eliminated because these topics are not the focus of this review, which looks into CP applications to problems with clinical relevance with an emphasis on predictive modeling. A paper using image features for forecasting was retained because the focus was predictive analytics and not image analysis. Similar searches performed in Google Scholar, PubMed, and IEEE Xplore did not provide any further work reporting the use of CP in medical applications.

## Conformal Prediction in Clinical Medical Sciences

The selection process described above gave a total of 14 papers reporting the use of CP in medical sciences with clinical relevance and germane to the topic of this review. These papers are listed in Table 1.

Pereira [14] and coworkers have used Conformal Prediction (CP) methods to predict confidence intervals of the probability that patients with mild cognitive impairment progress to dementia. In this work, the authors used two underlying classifiers, K-nearest neighbors (KNN) and Naïve Bayes, and Mondrian CP to evaluate the confidence of the predictions at different levels of significance. The methods were tested with two large available cohorts from prospective studies, the ADNI project (http://adni.loni.usc.edu/) [33] and the Cognitive Complains Cohort (CCC) [34]. The results show that the conformal predictors’ output regions contain the correct class within a precise level of confidence, but notice that better efficiency in the Mondrian steps is needed for clinical applications. The authors argue that the CP methods can help clinicians in making better use of AI methods in their practices. In a subsequent paper, the authors [20] compare CP predictors with Venn-ABERS predictors [35]. The authors use an ensemble classifier approach and compare the CP and Venn-ABERS confidence predictors with other direct probability estimates and other calibration methods given by standard classification methods. Using the same data sets that they used in ref. [14], the authors compare different combinations of classifiers and methods to predict the confidence of the prediction, concluding that different combinations and ensembles should be implemented depending on the intended use.

Papadopoulos [21, 22] and coworkers used CP to provide a measure of the accuracy of predictions of severe abdominal pain. The authors used a data set of 6,387 patients admitted to a hospital, for which 33 symptoms were recorded and coded into 135 binary attributes. These data were used to predict one of nine conditions that are associated with severe abdominal pain using a 2-layer fully connected feed-forward neural network (NN). The NN results were also compared with those from other classifiers demonstrating the NN performed better than other methods. The CP results using a Mondrian predictor show that at any confidence level, a matrix can be constructed to inform the probability of non-conformal predictions for each pair of conditions considered. The authors did not report any further studies evaluating the usefulness of the matrix in actual clinical environments. 

**Fig. 3.**
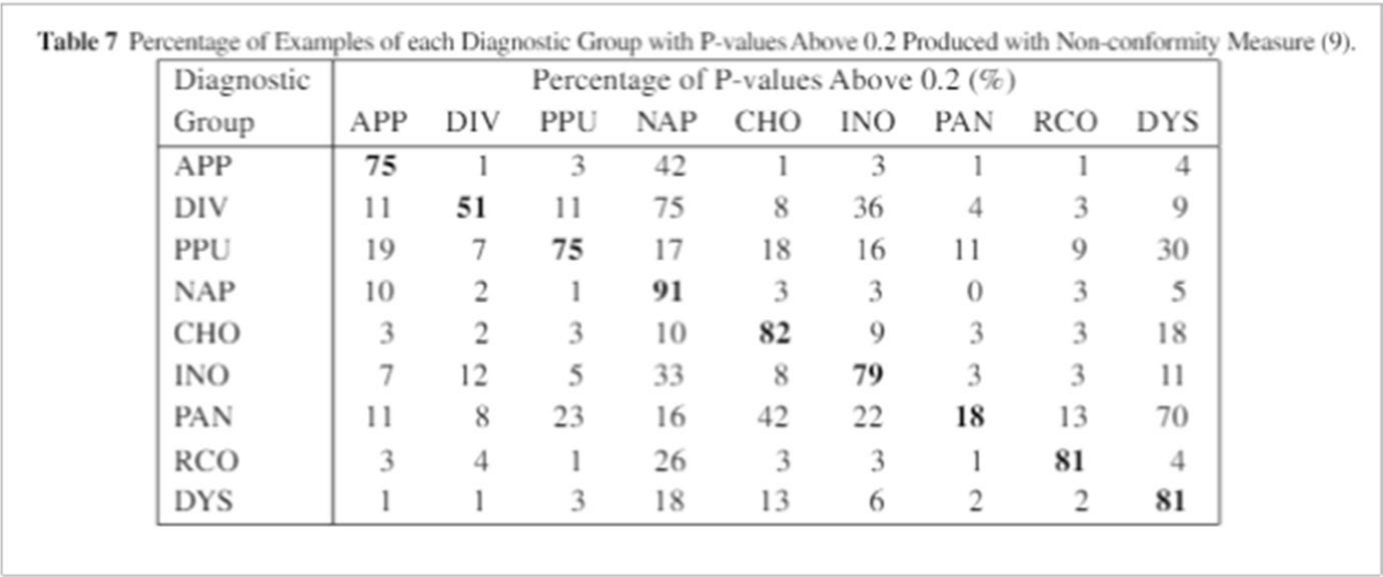
Example of the matrix that can be constructed to inform the probability of non-conformal predictions for each pair of conditions considered. From ref. [21]

Papadopoulos [23] also presented the use of CP to provide unbiased confidence measurements for stroke risk estimation based on ultrasound carotid images. In this work, the data from the ACSRS study [36] was used. The data set included 1,121 patients, for which 130 ipsilateral events were recorded. In addition to clinical and demographic variables, the study uses ten features extracted from the images. Using label-conditional Mondrian Conformal Prediction and artificial neural networks (ANNs), the authors compare several classification and CP approaches, concluding that the proposed LCMCP (Label-Conditional Mondrian Conformal Predictors) is superior to other approaches (Fig. 3).

Alnemer [24] reported the use of CP to assess the reliability of predictions of breast cancer survivability. The authors used the SEER cancer database [37] on which they applied several classification algorithms, including support vector machine (SVM), decision trees, K-nearest neighbors (KNN), and artificial neural networks (ANN), before evaluating the confidence of the prediction using CP to determine the non-conformity score and the confidence intervals, which were used to eliminate the non-reliable predictions. Using this approach, they consistently show that the CP corrected prediction always improved accuracy, sensitivity, specificity, and precision regardless of the classifier used.

Devetyarov [25] used conformal predictors to provide accuracy measurements of early diagnosis of ovarian and breast cancers using mass spectrometry data from the UKCTOCS biobank. The work uses a linear rule classifier with Mondrian predictors for CP. The results can provide information about the confidence and credibility of the predictions, as depicted in Table 1 of ref. [25], which clearly could be used as a base for the presentation of the results to practitioners.

In reference [26], the authors discuss how to incorporate a CP approach based on genetic algorithms (GA) and how to apply the method to predictions of breast cancer diagnosis using data from the Wisconsin breast cancer diagnosis (WBCD). They show that this approach to calculate CP is efficient and can provide similar results to other CP methods. In a subsequent paper, Lambrou and collaborators [27] used CP based on artificial neural networks (ANN), support vector machine (SVM), Naïve Bayes classifier (NBC), and K-nearest neighbor (KNN) classifiers to assess the reliability of predicting the risk of stroke based on morphological ultrasound images. The results show that the methods are useful to differentiate between symptomatic and asymptomatic plaques to assess the risk of stroke. Finally, the same authors [28] published a succinct review of using reliable confidence measurements for medical diagnosis with evolutionary algorithms, which recounts and expands the work described in their previous publications [26, 27].

Luo [29] introduced the concept of Dynamic CP (DCP) as part of a computer-aid decision support system for clinical decision-making using support vector machines (SVM) as the base classifier. The authors used their method on five non-clinical data sets and one clinical data set. The clinical data set is the MIT-BIT data set to detect arrhythmias [38]. The authors argue that their new DCP method provides multiple advantages over traditional CP in terms of computer performance and precision.

**Fig. 4.**
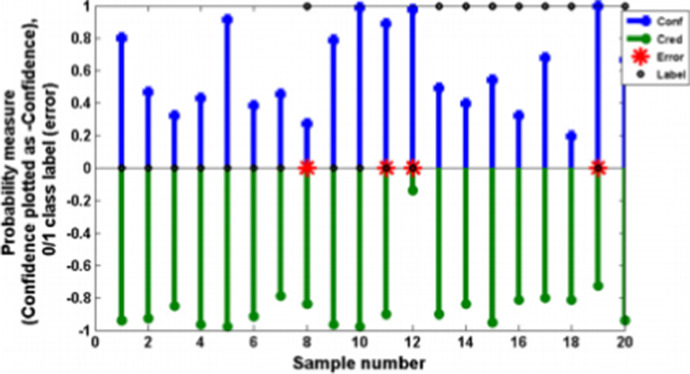
Visual representation of the Conformal Prediction results of the classification of mass spectroscopy traces used for cancer informatics in reference [30]

Schleif [30] used CP to obtain the reliability measurements of clinical measurements using mass spectroscopy when used for cancer informatics. The authors used a wavelet-based technique to encode the mass spectrometry signals from the clinical samples, using the results of the wavelet analysis as features for the classifiers. Clinical proteomic data for colorectal and lung cancer studies were used for this work. The features extracted by the wavelet process were classified using the supervised neural gas method, which combines the neural gas algorithm with the Generalized Learning Vector Quantizer [39]. An example of the results presented by the authors is given in Fig. 4, which demonstrates an interesting pictorial representation of the results that could be used to explain the results of the CP analysis to non-experts. The authors explain that using this figure, we can trust a prediction if the confidence is close to 100% and the credibility is not low (e.g., not less than 5%). Taking this advice into account, the results shown in the figure show that only items 4, 5, 9, 10, and 15 should be considered trusty results with high confidence and moderate or high credibility, and indeed, the labels for these items are correctly predicted.

Balasubramanian [31] applied CP to study the advantages that drug-eluting stents (DES) have over other percutaneous coronary intervention procedures, using a support vector machine (SVM) for the classification. The paper used a data set from Advanced Cardiac Specialists for patients in Arizona containing 2,312 patients who had a DES procedure during the period 2003–2007. The results of the analysis show that even at the 99% level of confidence, the number of empty predictions is very low, and argue that this approach can be very valuable in many predictive models in cardiology.

Finally, Bellotti [32] reported using CP to assess the reliability of the classification of childhood acute leukemia from gene expression data. In this work, a support vector machine (SVM) was used as the base classifier, and the authors show that the confidence machine proposed in the paper can be used to provide reliable predictions controlling the risk of error while maintaining the level of accuracy from the SVM.

## Conclusions

The literature reviewed here clearly shows that CP methods can be used in clinical applications and that they can provide important insight into the quality of individual predictions. The following CP methods have been used in clinical biomedical research: Inductive Conformal Predictors (ICP), Mondrian Conformal Predictors (MCP), Label-Conditional Mondrian Conformal Predictors (LCMCP), Dynamic Conformal Predictors (DCP), Inductive Confidence Machine (ICM), and Generalized Learning Vector Quantizer (GLVQ). This shows that there is interest in exploring the use of different CP approaches in biomedical sciences but that they have been used in a very diverse set of data sets, leaving unanswered the critical question of which are the best overall methods to be used across multiple clinical predictive tools and data sets. Studies using well-defined and commonly used analytic protocols in well-characterized data sets are needed to promote the use of CP in clinical settings.

Unfortunately, the review also shows that most of the studies have been performed in isolation and with little or no input from practicing clinicians, who should provide very important insights on how the results of CP assessments could be used in clinical practice.
